# α-Glucosidase, α-Amylase and Antioxidant Evaluations of Isolated Bioactives from Wild Strawberry

**DOI:** 10.3390/molecules27113444

**Published:** 2022-05-26

**Authors:** Mohammed A. Huneif, Seham M. Alqahtani, Alqahtani Abdulwahab, Sultan A. Almedhesh, Mater H. Mahnashi, Muhammad Riaz, Najm Ur-Rahman, Muhammad Saeed Jan, Farhat Ullah, Muhammad Aasim, Abdul Sadiq

**Affiliations:** 1 Pediatric Department, Medical College, Najran University, Najran 55461, Saudi Arabia; huneif@hotmail.com (M.A.H.); drseham2015@gmail.com (S.M.A.); aaalsharih@nu.edu.sa (A.A.); almedhesh31@hotmail.com (S.A.A.); 2 Department of Pharmaceutical Chemistry, College of Pharmacy, Najran University, Najran 55461, Saudi Arabia; 3 Department of Pharmacy, Shaheed Benazir Bhutto University, Sheringal 18050, Pakistan; pharmariaz@gmail.com (M.R.); najm@sbbu.edu.pk (N.U.-R.); 4 Department of Pharmacy, University of Swabi, Swabi 94640, Pakistan; saeedjanpharmacist@gmail.com; 5 Department of Pharmacy, Faculty of Biological Sciences, University of Malakand, Chakdara 18000, Pakistan; farhataziz80@hotmail.com; 6 Department of Biotechnology, Faculty of Biological Sciences, University of Malakand, Chakdara 18000, Pakistan; draasim@uom.edu.pk

**Keywords:** *Fragaria* *indica*, α-glucosidase, α-amylase, antioxidant, bioactives, antidiabetic

## Abstract

Diabetes mellitus is a metabolic disorder and is a global challenge to the current medicinal chemists and pharmacologists. This research has been designed to isolate and evaluate antidiabetic bioactives from *Fragaria indica*. The crude extracts, semi-purified and pure bioactives have been used in all in vitro assays. The in vitro α-glucosidase, α-amylase and DPPH free radical activities have been performed on all plant samples. The initial activities showed that ethyl acetate (**Fi.EtAc**) was the potent fraction in all the assays. This fraction was initially semi-purified to obtain **Fi.EtAc 1**–**3**. Among the semi-purified fractions, **Fi.EtAc 2** was dominant, exhibiting potent IC_50_ values in all the in vitro assays. Based on the potency and availability of materials, **Fi.EtAc 2** was subjected to further purification to obtain compounds **1** (2,4-dichloro-6-hydroxy-3,5-dimethoxytoluene) and **2** (2-methyl-6-(4-methylphenyl)-2-hepten-4-one). The two isolated compounds were characterized by mass and NMR analyses. The compounds **1** and **2** showed excellent inhibitions against α-glucosidase (21.45 for **1** and 15.03 for **2** μg/mL), α-amylase (17.65 and 16.56 μg/mL) and DPPH free radicals (7.62 and 14.30 μg/mL). Our study provides baseline research for the antidiabetic bioactives exploration from *Fragaria indica*. The bioactive compounds can be evaluated in animals-based antidiabetic activity in future.

## 1. Introduction

The use of plants or their derivatives to treat various ailments is a practice as old as human civilization [1,2]. These plants not only produce primary metabolites for their own existence but also release secondary metabolites to interact with other organisms. The secondary metabolites are usually investigated for various bioactives [3,4]. Bioactives are chemicals produced by a living being to treat various diseases or alter biological functions [5]. Over a span of decades, there have been attempts to explore the use of plants to treat various disorders, including diabetes [6,7].

Diabetes is a metabolic disorder characterized by an elevated level of blood glucose with common symptoms of polyphagia, polydipsia and polyurea [8]. There are several biochemical pathways which can be targeted for the management of diabetes [6]. The more trivial and vital enzymatic targets of diabetes are α-glucosidase, α-amylase, protein tyrosine phosphatase. The free radicals play a vital role in implications of several disorders [9,10,11]. In DM, the free radicals within the body increase due to auto-oxidation of glucose, which further complicates the situation. The free radicals damage the β cells, which are majorly responsible for the synthesis of insulin, and thus diminish the insulin synthesis [12]. The human body has the capability to combat the free radicals due to an auto-immune response [13]. However, at certain stages, when the free radicals are at uncontrolled level, the auto-immune system fails to control it [14]. At this stage of high free radicals within the body, the antioxidant treatment is necessary [15]. Reactive oxygen species and pro-inflammatory cytokines and chemokines cause the activation of JNK and NF-κB pathways that promote the development of diabetes [16]. There are 537 million adults suffering from diabetes and the number is expected to reach 783 million by 2045. The tremendous rise in diabetics confirms that it is a global challenge for policymakers and researchers to take necessary steps to overcome this challenge.

As the previous molecules are losing effectiveness, the result is certain effects, such as weight gain, heart problems, infections and weakened kidney. Despite significant additions to the list antidiabetics, researchers have to do more in search of safe, effective and efficient drug. Natural bioactives might be an effective therapeutic intervention, as studies have shown that phytochemicals are efficient agents in controlling diabetes via different mechanisms [17,18].

*Fragaria indica* Andrews, commonly known as wild strawberry, belongs to the family Rosaceae. It has scientific synonym *Duchesnea indica* (Andrews) and *Potentilla indica* (Andrews). It is naturalized in Africa, Europe and North America and distributed in Asian countries such as Pakistan, Kashmir, Afghanistan, Bhutan, China, India, Indonesia, Japan, Korea and Nepal [19]. The plant is known locally as *the Zmaki toot, Khunmurch,* and *Blmngye.* It is used traditionally for sore throat, tuberculosis, [20,21] nutrient, anthelmintic, and diabetic patients [22]. The plant has been explored for antioxidant, anti-inflammatory [23], and immunomodulatory potential [24,25]. Until now, other species of Fragaria such as *Fragaria × ananassa* and *Fragaria nilgerrensis* have been evaluated for their antidiabetic potentials [26,27]. However, to the best of our literature survey, we noticed that the antidiabetic potential of *Fragaria indica* is still unexplored. Keeping in view the global burden of diabetes and weaknesses of the available synthetic antidiabetic molecules and unexplored nature of *F. indica,* we plan the current work to investigate the antidiabetic and antioxidant potentials of the *F. indica* crude extract, semi-purified extracts and pure bioactives.

## 2. Results

### 2.1. Isolation of Bioactive Compounds

**Figure 1:**
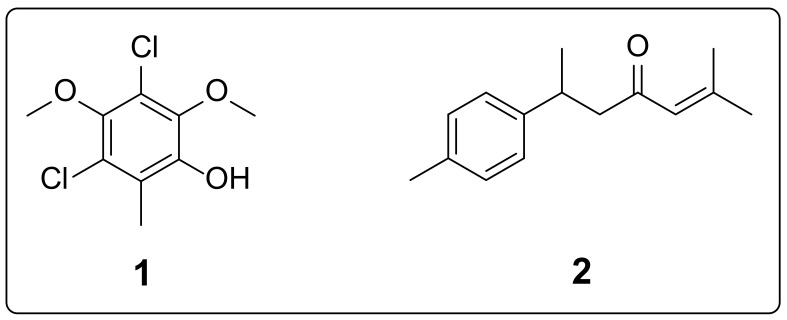
Isolated bioactive compounds from *Fragaria indica*.

In our designed study, we initially screened the crude extract and different solvent fractions of *F. indica* for in vitro α-glucosidase, α-amylase and DPPH activities. Based on the potency of ethyl acetate fraction (**Fi.EtAc**), we subjected the ethyl acetate fraction to normal gravity column chromatography with slow elution of solvent system. The solvent system was started with 100% *n*-hexane and was gradually increased in polarity by adding small percentages of ethyl acetate. Initially, we obtained three semi-purified phytocomponents **Fi.EtAc 1**, **Fi.EtAc 2** and **Fi.EtAc 3**. The semi-purified phytochemicals were further subjected to in vitro α-glucosidase, α-amylase and DPPH activities. The semi-purified components were isolated based on TLC analysis. Among the three semi-purified ethyl acetate fractions, **Fi.EtAc 2** was the potent one based on its IC_50_ values obtained in the in vitro assays. The **Fi.EtAc 2** was further purified to obtain two bioactives **1** and **2** (as shown in Figure 1).

### 2.2. Structure Confirmations of the Isolated Bioactives

The compound **1** is chemically 2,4-dichloro-6-hydroxy-3,5-dimethoxytoluene. The molecular weight of compound **1** was confirmed as 236 [M − 1]^+^, and its fragmentation pattern was 236 (68%, molecular ion peak), 221 (100%, the base peak), 206 (2%), 193 (25%), 178 (38%), 171 (5%), 158 (15%), 143 (14%), 127 (5%), 103 (11%), 87 (19%) and 67 (18%). The ^1^H-NMR spectrum of compound **1** showed three distinct singlets, each of 3H. The toluoyl methyl group (Ar-CH_3_) appeared at 2.314 chemical shift. The two methoxy groups (CH_3_O-Ar-OCH_3_) appeared as two singlets at 3.870 and 3.877 chemical shift. The phenolic hydrogen (HO-Ar) appeared as singlet of one proton at 6.041. The ^13^C-NMR of compound **1** showed signals at chemical shift values of 15.38, 55.03, 112.18, 115.70, 119.75, 133.11, 137.39 and 139.59 ppm.

Chemically, compound **2** is 2-methyl-6-(4-methylphenyl)-2-hepten-4-one. The molecular weight of compound **2** was confirmed as 216, and its fragmentation pattern was 216 (28%, molecular ion peak), 201 (10%), 132 (16%), 119 (46%), 83 (100%, base peak) and 55 (25%). The ^1^H-NMR spectrum also showed all the proton patterns of the compound **2**. The four aromatic protons appeared in two distinct doublets (each having two protons integration) at 7.12 and 7.07 chemical shift values. The observed coupling constant values (*J*) were 8.36 and 8.05 Hz, respectively for the two doublets. The hydrogen atom on the alkene unit of the compound **2** appeared at 6.151 as a sharp singlet. One of the methylene protons of compound **2** (-CH_2_-) gave a multiplet between chemical shift of 3.463 and 3.373. The second methylene proton (-CH_2_-) merged with methyne proton (-CH-) in the multiplet range of 3.054 to 2.963. The aromatic methyl group (Ar-CH_3_) appeared as a singlet of three protons at 2.238. The two methyl groups attached with the alkene unit gave two distinct singlets at 2.082 and 1.994, with integration values of 3H for each one. The methyl group attached at α-position to the aromatic ring gave a doublet at 1.28 with a *J* value of 7.07 Hz. The ^13^C-NMR of compound **2** showed signals at chemical shift values of 18.03, 19.45, 21.78, 26.21, 37.67, 45.83, 126.92, 130.60, 132.20, 137.16, 140.14, 156.05 and 201.24 ppm. The mass and NMR spectra of the isolated bioactive compounds are provided in the Supplementary Materials.

### 2.3. In Vitro Activities on Crude Extracts of Fragaria indica

#### 2.3.1. α-Glucosidase and α-Amylase

The α-glucosidase and α-amylase activities of the crude extracts and subfractions of *Fragaria indica* are summarized in Table 1. The tested concentrations of the plant’s samples and standard drug used were 1000, 500, 250, 125 and 62.5 μg/mL. At maximum concentrations, the crude extract (**Fi.Cr**), *n*-hexane (**Fi.Hex**), ethyl acetate (**Fi.EtAc**), chloroform (**Fi.Chf**) and aqueous (**Fi.Aq**) fractions gave percent inhibitions of 64.44, 61.50, 71.69, 61.23 and 57.85%. The standard drug acarbose at the same concentration (1000 μg/mL) gave a percent inhibition of 96.00%. Overall in our fractions, **Fi.EtAc** was the potent one in inhibition of α-glucosidase enzyme with the observation of an IC_50_ value of 117.54 μg/mL.

Likewise, the α-amylase inhibitory potential was also in a similar pattern to that of α-glucosidase, as shown in Table 1. The observed IC_50_ values for **Fi.Cr**, **Fi.Hex**, **Fi.EtAc**, **Fi.Chf** and **Fi.Aq** fractions were 218.19, 249.85, 96.82, 259.11 and 398.46 μg/mL, respectively in comparison to the standard acarbose (12.84 μg/mL). In α-glucosidase and α-amylase activities, we observed that ethyl acetate fraction of *Fragaria indica* (**Fi.EtAc**) was the potent inhibitor fraction.

#### 2.3.2. DPPH Results

The antioxidant potentials of crude extracts and different solvent fractions of *Fragaria inidica* were assessed by DPPH assay and the results are shown in Table 2. Based on Table 2, we can clearly observe that overall, the plant contains potential antioxidant properties. At 1000 μg/mL, **Fi.Cr**, **Fi.Hex**, **Fi.EtAc**, **Fi.Chf** and **Fi.Aq** fractions gave 65.66, 63.44, 78.81, 67.85 and 60.54% inhibitions. Ascorbic acid gave 91.90% inhibition at the highest tested concentration under the same set of experiments. In the DPPH assay, the **Fi.EtAc** was the fraction with potent IC_50_ value of 59.55 μg/mL in comparison to the standard drug IC_50_ value, which was noted as 4.98 μg/mL. The other fractions showed IC_50_ values of 200.89 (**Fi.Cr**), 236.91 (**Fi.Hex**), 142.39 (**Fi.Chf**) and 349.35 μg/mL (**Fi.Aq**).

### 2.4. In Vitro Activities on Semi-Purified Ethyl Acetate Fractions of Fragaria indica

#### 2.4.1. In Vitro α-Glucosidase and Amylase Results

The α-glucosidase and α-inhibitory potentials of semi-purified ethyl acetate fractions (**Fi.EtAc 1**, **Fi.EtAc 2** and **Fi.EtAc 3**) are shown in Table 3. As obvious from Table 3 results, the activities profile enhanced with the semi-purification. Specifically, the semi-purified ethyl acetate fraction **Fi.EtAc 2** exhibited IC_50_ values in parallel comparison to the standard drug. The fraction **Fi.EtAc 2** exhibited IC_50_ values of 14.81 and 14.54 μg/mL against α-glucosidase and α-amylase enzymes, respectively. The standard drug gave IC_50_ values of 17.28 and 12.84 μg/mL against α-glucosidase and α-amylase enzymes, respectively. Similarly, the potencies of other semi-purified fractions were also excellent. The fraction **Fi.EtAc 1** gave IC_50_ values of 63.85 and 38.60 μg/mL against α-glucosidase and α-amylase enzymes, respectively. Similarly, the semi-purified fraction **Fi.EtAc 3** gave IC_50_ values of 20.56 μg/mL (α-glucosidase) and 19.62 μg/mL (α-amylase).

#### 2.4.2. DPPH Results

The three semi-purified ethyl acetate fractions of *Fragaria indica* were also found to possess strong antioxidant properties, as can be seen in Table 4. With semi-purification, the fractions showed excellent antioxidant activity profiles. The observed IC_50_ values for the three fractions **Fi.EtAc 1**, **Fi.EtAc 2** and **Fi.EtAc 3** were 14.95, 20.59 and 26.25 μg/mL, respectively in DPPH free radicals scavenging activity. Based on the relative potencies and amount of each semi-purified fraction, we selected **Fi.EtAc 2** for further purification to obtain the bioactive compounds.

### 2.5. In Vitro Activities on Isolated Compounds of Fragaria indica

The two isolated compounds were characterized and were then subjected to in vitro antidiabetic activities and results are summarized in Table 5. The compound **1** (2,4-dichloro-6-hydroxy-3,5-dimethoxytoluene) and **2** (2-methyl-6-(4-methylphenyl)-2-hepten-4-one) showed excellent activities profiles exhibiting IC_50_ values of 21.45 and 17.65 (α-glucosidase) and 15.03 and 16.56 μg/mL (α-amylase), respectively. Similarly, of the two compounds, specifically, the phenolic type of derivative (**1**) was the more potent antioxidant. The observed IC_50_ values for compounds **1** and **2** were 7.62 and 14.30 μg/mL, respectively against the DPPH free radicals. The standard drug IC_50_ under the same set of condition was 4.98 μg/mL.

## 3. Discussion

Researchers, specifically the medicinal chemists and pharmacologists are constantly searching for new drug molecules [28,29,30]. The organic chemists design and explore new methods for the synthesis of valuable molecules which can be important drug or natural products [31,32]. On the other hand, the medicinal chemists are in constant evaluation of medicinal compounds for various vital targets [33,34,35]. The pharmacological researchers use various in vitro and in vivo methods to confirm the possible use of a compound for a specific disease [36]. One of the major tools is the computational approach, which allows researchers to obtain the binding energies for a specific biological target [37]. The medicinal values of compounds can be explored from natural and synthetic origins [38,39]. Among the major health issues, diabetes is a global challenge to the researchers. Various natural and synthetic origins have been employed for the management of diabetes [40]. However, due to the emerging increasing number of diabetes patients, more natural sources can be explored to treat diabetes. Poly- and oligosaccharides are converted into monosaccharides by the action of intestinal α-glucosidase and α-amylase, and the activity significantly contributes to postprandial hyperglycemia. Synthetic agents such as acarbose can inhibit these enzymes, but the side effects, such as diarrhea, flatulence, and stomach pain, make them unsuitable [41]. The present study was designed to develop antidiabetics from natural products based on this strategy with lesser side effects [42].

The ethyl acetate fraction of *F. indica* showed maximum inhibition of α-glucosidase, α-amylase enzyme and DPPH free radical among the tested fractions and crude extract. The inhibitory activity was enhanced with semi-purification of the ethyl acetate fraction. The isolated compounds showed equivalent potent activities to the standard used.

Compound **1** is chemically 2,4-dichloro-6-hydroxy-3,5-dimethoxytoluene, while compound **2** is 2-methyl-6-(4-methylphenyl)-2-hepten-4-one. Compound **1** seems to be the derivative of 3,5-dimethoxytoluene, an important aromatic compound of roses with sedative action and used in aromatherapy as a relaxing fragrance [43]. Compound **2**, commonly named ar-turmerone, was isolated from *Curcuma longa* with proven antivenom and anti-inflammatory potentials [44,45]. It is a major component of turmeric oil with anti-dermatophytic activity [46] and a neuroprotective effect [47]. However, the first time it was reported was in *F. indica*.

The wild-type strawberry, i.e., *F. indica* herb was investigated for the first time for in vitro antidiabetic and antioxidant activities. However, other species, especially fruits, are extensively investigated for such activities. The enzyme α-amylase and α-glucosidase activity were inhibited by ellagic acid or derivatives in aqueous fruit extract of *Fragaria* × a*nanassa* [26]. Similarly, another specie *F. vesca* leaf extract was reported to inhibit α-glucosidase and α-amylase enzyme activity [41]. Flavonoids in *n*-butanol extract of *F. nilgerrensis* Schlecht produced an antidiabetic effect [27].

The antioxidant properties of strawberries have been well documented, both in vitro and in vivo [48,49,50]. A study on aqueous extract of *Fragaria vesca* L. collected in Bulgaria showed strong ABTS inhibitory activity due to more polyphenols than those with less ABTS inhibitory potentials [51].

Strawberries and strawberry-based phytochemicals are potential dietary sources for managing hyperglycemia [52]. The leaves of the *Fragaria* × *ananassa* Duch. cultivars have also been reported for antihyperglycemic and antioxidant potentials [53].

The beneficial effects of the different strawberry extracts on blood glucose levels are popularizing its role as functional food [54,55]. The role was further supported by a clinical trial where a diet supplemented with strawberry fruit extract lowered glycohemoglobin (HbA1c) levels after six weeks of treatment [56].

Our experiments revealed that polyphenolic compounds of ethyl acetate fractions, e.g., compounds **1** and **2**, are potential major components possessing anti-diabetic and antioxidant potentials; however, further analytical and structure-activity studies are required to identify and synthesize more active components in the field.

## 4. Materials and Methods

### 4.1. Collection of Medicinal Plant and Extraction

The plant was collected from Laram Qilla Talash (Latitude 45.990530, Longitude 12.673570), District Dir Lower of Khyber Pakhtunkhwa (KPK) Pakistan in August 2020 and was identified as *Fragaria indica* Andrews by Nasrullah, Plant Taxonomist at the department of Botany University of Malakand, Chakdara, Pakistan. A sample specimen was deposited at the herbarium of the same department, voucher no. H.UOM.BG.551. The extraction was carried out as per our previous mentioned procedure [57]. The crude extract was subjected to different solvents fractions based on the polarity basis as per our previously reported procedure [58].

### 4.2. Phytochemistry and Bioactives Isolation

In our initial in vitro studies, we observed that among all the fractions, ethyl acetate fraction (**Fi.EtAc**) was the most prominent. Based on this, the ethyl acetate fraction was subjected to gravity column. The column elution was started with 100% of pure *n*-hexane as non-polar component. The polarity of solvent was gradually increased with the addition of ethyl acetate as polar modifier solvent. After the column chromatography, we were able to collect three different ethyl acetate fractions (**Fi.EtAc 1**–**3**) as visualized on pre-coated TLC plate under UV lamp. The three semi-purified ethyl acetate fractions were also subjected to all in vitro assays. Among the semi-purified ethyl acetate fractions, **Fi.EtAc 2** was observed with potent IC_50_ values in our in vitro experiments. Based on the potency, **Fi.EtAc 2** was further subjected to purification to obtain the bioactive compounds. At the end of purification process, we were able to obtain two bioactive compounds (**1** and **2**). The isolated compounds were characterized by their masses and also their structures were confirmed by proton NMR [6].

### 4.3. In Vitro α-Glucosidase Inhibition

*α*-Glucosidase inhibitory assay on *F. indica* extracts, solvent fractions and isolated compounds were performed according to the reported procedure [59] using acarbose as standard drug. The *α*-glucosidase solution (120 μL) of 0.50 U/mL in 0.10 M phosphate buffer (pH 6.90) was prepared and *p*-nitrophenyl-*α*-d-glucopyranoside (5 mM) was added as substrate solution. Different concentrations ranging from 62.50 µg/mL to 1000 µg/mL of the crude extracts, semi-purified fractions and isolated compounds of *F. indica* were prepared accordingly. The plant’s samples (extracts, semi-purified and purified compounds) were mixed with the solutions of enzyme as per the experiment and were incubated at 37 °C.

After initial incubation for 15 min, 20 μL *p*-nitrophenyl-*α*-d-glucopyranoside solution was mixed and was again incubated in the same way. Finally, 80 μL solution of sod. carbonate (0.20 M) was added to finish up the reaction. The solution containing all the substrates except α-glucosidase served as a blank. The absorbances of experiments were measured at 405 nm. The experiments were repeated 3 times and the calculations were made as per the standard protocols.

### 4.4. In Vitro α-Amylase Inhibition

The *α*-amylase inhibitory activity was carried out according to the established protocols for *F. indica* extracts and isolated compounds [60] The amylase solution was prepared in phosphate buffer. The solutions of the *F. indica* and isolated compounds (62.50 to 1000 µg/mL) were added to the amylase solution and allowed to react. After reaction completion, starch solution was added and incubated as per the standard method. Afterwards, a solution of dinitro-salicylic acid was added to both test and control groups. The final solutions were heated for 3 min in boiling water and the absorbances were measured at 656 nm. The experiments were performed in triplicates and percent inhibitions were calculated.

### 4.5. DPPH Free Radicals Scavenging Assay

The antioxidant activity was carried out for *F. indica* extracts, semi-purified ethyl acetate fractions and isolated compounds using DPPH free radicals. DPPH solution was prepared (24 mg/100 mL of methanol). Stock solutions (1 mg/mL) of *F. indica* extracts and isolated compounds were prepared in methanol and then diluted to 1000, 500, 250, 125, 62.5 μg/mL. Sample and DPPH solutions were mixed in a ratio of 1:1 and were incubated at 23 °C for 30 min. Finally, absorbance was measured at 517 nm using UV spectrophotometer (Thermo electron corporation, Waltham, MA, USA). Ascorbic acid was used as positive control. Percent radical scavenging activity was measured using the reported equations [61].

### 4.6. Estimation of IC_50_ Values

The half inhibitory concentration (IC_50_) was calculated using Microsoft Excel program as per our previously reported method [62].

### 4.7. Statistical Data Analysis

The results in assays were expressed as mean ± S.E.M. GraphPad prism (GraphPad Software, San Diego, CA, USA) was used for one-way ANOVA followed by Dunnett’s multiple comparison test with positive control and test groups. The *p* values less than 0.05 were considered as statistically significant. The level of significance was expressed as * = *p* < 0.05, ** = *p* < 0.01 and *** = *p* < 0.001.

## 5. Conclusions

It can be concluded from our current research results that *Fragaria indica* is a rich source of bioactives. Herein, we have explored the *Fragaria indica* for its antidiabetic potential. We initially confirmed from crude extracts’ in vitro assays that ethyl acetate fraction of the wild strawberry was potent among all. Then, we semi-purified ethyl acetate fractions **Fi.EtAc 1** to **3**. The **Fi.EtAc 2** was the fraction with dominant activities. We were able to isolate two compounds, i.e., **1** (2,4-dichloro-6-hydroxy-3,5-dimethoxytoluene) and **2** (2-methyl-6-(4-methylphenyl)-2-hepten-4-one). The two compounds showed overwhelming activity profile against α-glucosidase, α-amylase and DPPH free radicals. The two compounds can be further evaluated for antidiabetic targets using in vivo models to obtain more fruitful results.

## Figures and Tables

**Table 1:** α-Glucosidase and α-amylase results of *Fragaria indica* crude extracts.

Sample	Concentration (μg/mL)	α-Glucosidase		α-Amylase	
		Percent Inhibition	IC_50_ (μg/mL)	Percent Inhibition	IC_50_ (μg/mL)
**Fi.Cr**	1000	64.44 ± 0.09 ***	232.10	66.90 ± 0.72 ***	218.19
	500	56.87 ± 0.39 ***		57.12 ± 0.89 ***	
	250	51.83 ± 1.07 ***		52.64 ± 1.38 ***	
	125	44.23 ± 0.44 ***		45.40 ± 0.93 ***	
	62.5	36.29 ± 0.43 ***		35.22 ± 0.94 ***	
**Fi.Hex**	1000	61.50 ± 2.26 ***	340.56	65.50 ± 2.26 ***	249.85
	500	54.01 ± 0.42 ***		56.01 ± 0.42 ***	
	250	45.07 ± 0.62 ***		49.07 ± 0.62 ***	
	125	39.70 ± 0.35 ***		43.70 ± 0.35 ***	
	62.5	34.73 ± 0.66 ***		35.73 ± 0.66 ***	
**Fi.EtAc**	1000	71.69 ± 0.77 ***	117.54	73.60 ± 1.63 ***	96.82
	500	63.67 ± 0.61 ***		66.82 ± 0.85 ***	
	250	56.44 ± 0.51 ***		61.25 ± 1.40 ***	
	125	51.76 ± 0.58 ***		53.10 ± 0.60 ***	
	62.5	43.54 ± 0.50 ***		44.61 ± 0.43 ***	
**Fi.Chf**	1000	61.23 ± 0.22 ***	296.86	63.48 ± 0.25 ***	259.11
	500	55.45 ± 0.90 ***		56.47 ± 0.04 ***	
	250	46.90 ± 0.60 ***		48.47 ± 0.44 ***	
	125	41.00 ± 0.30 ***		42.44 ± 0.09 ***	
	62.5	37.90 ± 0.45 ***		37.43 ± 1.39 ***	
**Fi.Aq**	1000	57.85 ± 0.56 ***	429.39	57.79 ± 0.62 ***	398.46
	500	51.64 ± 0.75 ***		52.45 ± 0.49 ***	
	250	44.58 ± 0.77 ***		46.75 ± 0.58 ***	
	125	38.75 ± 0.63 ***		38.73 ± 0.64 ***	
	62.5	32.58 ± 0.70 ***		33.47 ± 0.56 ***	
Acarbose	1000	96.00 ± 0.30	17.28	82.43 ± 0.52	12.84
	500	90.61 ± 0.43		74.03 ± 0.64	
	250	84.03 ± 0.86		71.56 ± 0.49	
	125	77.58 ± 0.77		67.05 ± 0.49	
	62.5	71.48 ± 0.74		63.26 ± 0.93	

All values are taken as mean ± SEM (*n* = 3). Two-way ANOVA followed by Bonferoni test were followed. Values significantly different in comparison to standard drug, i.e., *** = *p* < 0.001.

**Table 2:** DPPH free radicals scavenging results of crude extracts of *Fragaria indica*.

Sample	Concentration (μg/mL)	Percent Inhibition	IC_50_ (μg/mL)
**Fi.Cr**	1000	65.66 ± 0.78 ***	200.89
	500	60.62 ± 0.74 ***	
	250	52.62 ± 0.74 ***	
	125	44.86 ± 0.60 ***	
	62.5	37.48 ± 0.64 ***	
**Fi.Hex**	1000	63.44 ± 0.09 ***	236.91
	500	57.87 ± 0.39 ***	
	250	51.83 ± 1.07 ***	
	125	43.23 ± 0.44 ***	
	62.5	36.29 ± 0.43 ***	
**Fi.EtAc**	1000	76.81 ± 0.60 ***	59.55
	500	70.74 ± 0.61 ***	
	250	64.68 ± 0.60 ***	
	125	58.63 ± 0.76 ***	
	62.5	49.79 ± 0.63 ***	
**Fi.Chf**	1000	67.85 ± 0.56 ***	142.39
	500	62.64 ± 0.75 ***	
	250	55.58 ± 0.77 ***	
	125	47.75 ± 0.63 ***	
	62.5	42.58 ± 0.70 ***	
**Fi.Aq**	1000	60.54 ± 0.48 ***	349.35
	500	52.30 ± 0.66 ***	
	250	45.58 ± 0.59 ***	
	125	41.52 ± 0.62 ***	
	62.5	35.45 ± 0.57 ***	
Ascorbic acid	1000	91.90 ± 0.96	4.98
	500	87.08 ± 0.47	
	250	82.40 ± 0.20	
	125	77.61 ± 0.43	
	62.5	75.45 ± 0.90	

All values are taken as mean ± SEM (*n* = 3). Two-way ANOVA followed by Bonferoni test were followed. Values significantly different in comparison to standard drug, i.e., *** = *p* < 0.001.

**Table 3:** α-Glucosidase and α-amylase results of semi-purified ethyl acetate fraction of *Fragaria indica*.

Sample	Concentration (μg/mL)	α-Glucosidase		α-Amylase	
		Percent Inhibition	IC_50_ (μg/mL)	Percent Inhibition	IC_50_ (μg/mL)
**Fi.EtAc 1**	1000	81.81 ± 0.60 ***	63.85	82.45 ± 0.55 ^ns^	38.60
	500	76.74 ± 0.61 ***		76.53 ± 0.41 ^ns^	
	250	67.68 ± 0.60 ***		71.42 ± 0.46 ^ns^	
	125	61.63 ± 0.76 ***		65.68 ± 0.64 *	
	62.5	47.79 ± 0.63 ***		53.63 ± 0.64 ***	
**Fi.EtAc 2**	1000	87.63 ± 0.64 ***	14.81	89.37 ± 0.54 ^ns^	14.54
	500	82.45 ± 0.55 ***		84.44 ± 0.50 ^ns^	
	250	76.53 ± 0.41 ***		77.51 ± 0.72 ^ns^	
	125	71.42 ± 0.46 ***		72.28 ± 0.61 ^ns^	
	62.5	65.68 ± 0.64 ***		67.46 ± 0.62 ^ns^	
**Fi.EtAc 3**	1000	83.08 ± 1.04 ***	20.56	85.43 ± 1.26 ^ns^	19.62
	500	76.45 ± 0.90 ***		78.83 ± 0.66 ^ns^	
	250	70.58 ± 0.63 ***		72.93 ± 0.90 ^ns^	
	125	65.40 ± 0.20 ***		67.26 ± 0.77 ^ns^	
	62.5	61.80 ± 0.90 ***		63.10 ± 0.95 ^ns^	
Acarbose	1000	96.00 ± 0.30	17.28	82.43 ± 0.52	12.84
	500	90.61 ± 0.43		74.03 ± 0.64	
	250	84.03 ± 0.86		71.56 ± 0.49	
	125	77.58 ± 0.77		67.05 ± 0.49	
	62.5	71.48 ± 0.74		63.26 ± 0.93	

All values are taken as mean ± SEM (*n* = 3). Two-way ANOVA followed by Bonferoni test were followed. Values significantly different in comparison to standard drug, i.e., * = *p* < 0.05, *** = *p* < 0.001 and ns: not significant.

**Table 4:** DPPH results of semi-purified ethyl acetate fractions of *Fragaria indica*.

Sample	Concentration (μg/mL)	Percent Inhibition	IC_50_ (μg/mL)
**Fi.EtAc 1**	1000	95.00 ± 0.32 ^ns^	14.95
	500	90.63 ± 0.45 ^ns^	
	250	84.05 ± 0.88 ^ns^	
	125	78.56 ± 0.79 ^ns^	
	62.5	71.46 ± 0.76 **	
**Fi.EtAc 2**	1000	85.72 ± 0.79 ***	20.59
	500	81.68 ± 0.63 ***	
	250	76.46 ± 0.53 ***	
	125	69.78 ± 0.60 ***	
	62.5	61.56 ± 0.52 ***	
**Fi.EtAc 3**	1000	84.83 ± 0.62 ***	26.25
	500	80.76 ± 0.63 ***	
	250	75.70 ± 0.62 ***	
	125	66.65 ± 0.78 ***	
	62.5	59.81 ± 0.65 ***	
Ascorbic acid	1000	91.90 ± 0.96	4.98
	500	87.08 ± 0.47	
	250	82.40 ± 0.20	
	125	77.61 ± 0.43	
	62.5	75.45 ± 0.90	

All values are taken as mean ± SEM (*n* = 3). Two-way ANOVA followed by Bonferoni test were followed. Values significantly different in comparison to standard drug, i.e., ** = *p* < 0.01, *** = *p* < 0.001 and ns: not significant.

**Table 5:** α-Glucosidase, α-amylase and DPPH results of isolated compounds from *Fragaria indica*.

Sample	α-Glucosidase IC_50_ (μg/mL)	α-Amylase IC_50_ (μg/mL)	DPPH IC_50_ (μg/mL)
Compound **1**	21.45	17.65	7.62
Compound **2**	15.03	16.56	14.30
Acarbose	17.28	12.84	-
Ascorbic acid	-	-	4.98

## Data Availability

The whole data is available within the manuscript and the Supplementary Materials.

## Supplementary Materials

The following are available online at https://www.mdpi.com/article/10.3390/molecules27113444/s1. The NMR and MS spectra are provided in the supporting information. Figure S1: GC-MS chromatogram of Compound **1**; Figure S2: ^1^H-NMR spectrum of Compound **1**; Figure S3: ^13^C-NMR spectrum of Compound **1**; Figure S4: GC-MS chromatogram of Compound **2**; Figure S5: ^1^H-NMR spectrum of Compound **2**; Figure S6: ^13^C-NMR spectrum of Compound **2**.

## Sample Availability

Samples of the plant are available from the authors.

## References

[1] Süntar I. (2019). Importance of ethnopharmacological studies in drug discovery: Role of medicinal plants. Phytochem. Rev..

[2] Akram M., Tahir I.M., Shah S.M.A., Mahmood Z., Altaf A., Ahmad K., Munir N., Daniyal M., Nasir S., Mehboob H. (2018). Antiviral potential of medicinal plants against HIV, HSV, influenza, hepatitis, and coxsackievirus: A systematic review. Phytother. Res..

[3] Verpoorte R. (1998). Exploration of nature’s chemodiversity: The role of secondary metabolites as leads in drug development. Drug Discov. Today.

[4] Zafar R., Ullah H., Zahoor M., Sadiq A. (2019). Isolation of bioactive compounds from *Bergenia ciliata* (haw.) Sternb rhizome and their antioxidant and anticholinesterase activities. BMC Complement. Altern. Med..

[5] Ksean M. (2012). Natural products research. Nat. Prod. Chem. Res..

[6] Mahnashi M.H., Alqahtani Y.S., Alqarni A.O., Alyami B.A., Jan M.S., Ayaz M., Ullah F., Rashid U., Sadiq A. (2021). Crude extract and isolated bioactive compounds from *Notholirion thomsonianum* (Royale) Stapf as multitargets antidiabetic agents: In-vitro and molecular docking approaches. BMC Complement. Med. Ther..

[7] Sadiq A., Rashid U., Ahmad S., Zahoor M., Alajmi M.F., Ullah R., Noman O.M., Ullah F., Ayaz M., Khan I. (2020). Treating Hyperglycemia From *Eryngium caeruleum* M. Bieb: In-vitro α-Glucosidase, Antioxidant, in-vivo Antidiabetic and Molecular Docking-Based Approaches. Front. Chem..

[8] Care D. (2018). Economic Costs of Diabetes in the US in 2017. Diabetes Care.

[9] Shah S.M.M., Sadiq A., Ullah F., Shah S.M.H. (2014). Antioxidant, total phenolic contents and antinociceptive potential of *Teucrium stocksianum* methanolic extract in different animal models. BMC Complement. Altern. Med..

[10] Sadiq A., Mahmood F., Ullah F., Ayaz M., Ahmad S., Haq F.U., Khan G., Jan M.S. (2015). Synthesis, anticholinesterase and antioxidant potentials of ketoesters derivatives of succinimides: A possible role in the management of Alzheimer’s. Chem. Central J..

[11] Zafar R., Zubair M., Ali S., Shahid K., Waseem W., Naureen H., Haider A., Jan M.S., Ullah F., Sirajuddin M. (2021). Zinc metal carboxylates as potential anti-Alzheimer’s candidate: In vitro anticholinesterase, antioxidant and molecular docking studies. J. Biomol. Struct. Dyn..

[12] Hussain F., Khan Z., Jan M.S., Ahmad S., Ahmad A., Rashid U., Ullah F., Ayaz M., Sadiq A. (2019). Synthesis, in-vitro α-glucosidase inhibition, antioxidant, in-vivo antidiabetic and molecular docking studies of pyrrolidine-2,5-dione and thiazolidine-2,4-dione derivatives. Bioorg. Chem..

[13] Puertollano M.A., Puertollano E., De Cienfuegos G.A., De Pablo M.A. (2011). Dietary Antioxidants: Immunity and Host Defense. Curr. Top. Med. Chem..

[14] Zeb A., Sadiq A., Ullah F., Ahmad S., Ayaz M. (2014). Investigations of anticholinestrase and antioxidant potentials of methanolic extract, subsequent fractions, crude saponins and flavonoids isolated from *Isodon rugosus*. Biol. Res..

[15] Bibi A., Shah T., Sadiq A., Khalid N., Ullah F., Iqbal A. (2019). l-Isoleucine-catalyzed Michael Synthesis of *N*-Alkylsuccinimide Derivatives and Their Antioxidant Activity Assessment. Russ. J. Org. Chem..

[16] Shoelson S.E., Lee J., Goldfine A.B. (2006). Inflammation and insulin resistance. J. Clin. Investig..

[17] Dembinska-Kiec A., Mykkänen O., Kiec-Wilk B., Mykkänen H. (2008). Antioxidant phytochemicals against type 2 diabetes. Br. J. Nutr..

[18] Leiherer A., Mündlein A., Drexel H. (2013). Phytochemicals and their impact on adipose tissue inflammation and diabetes. Vasc. Pharmacol..

[19] Qureshi H., Arshad M., Bibi Y. (2014). Invasive flora of Pakistan: A critical analysis. Int. J. Biosci..

[20] Kayani S., Ahmad M., Zafar M., Sultana S., Khan M.P.Z., Ashraf M.A., Hussain J., Yaseen G. (2014). Ethnobotanical uses of medicinal plants for respiratory disorders among the inhabitants of Gallies—Abbottabad, Northern Pakistan. J. Ethnopharmacol..

[21] Hamayun M., Khan M.A., Begum S. (2003). Marketing of medicinal plants of Utror-Gabral valleys, Swat, Pakistan. Ethnobot Leafl..

[22] Saqib A.A., Gul S. (2018). Traditional knowledge of medicinal herbs among indigenous communities in Maidan Valley, Lower Dir, Pakistan. Bull. Environ. Pharmacol. Life Sci..

[23] Sereno-Villaseñor L., Hernández-García A., Torres-Martínez R., Meléndez-Herrera E., Manzo-Avalos S., Martínez-Flores H.E., Saavedra-Molina A., Salgado-Garciglia R. (2020). Antioxidant and Anti-inflammatory Effects of the Methanolic Extract of *Potentilla indica* Fruits. FASEB J..

[24] Ang H.Y., Subramani T., Yeap S.K., Omar A.R., Ho W.Y., Abdullah M.P., Alitheen N.B. (2014). Immunomodulatory effects of *Potentilla indica* and *Dendrophthoe pentandra* on mice splenocytes and thymocytes. Exp. Ther. Med..

[25] Long M., Yu X., Li B., Xiong Y., Xiang B., He Q. (2020). Characterisation of antioxidant, anti-inflammatory, and immunomodulatory activities of polysaccharides derived from *Duchesnea indica* (Andrews) Focke. Int. Food Res. J..

[26] Pinto M.D.S., de Carvalho J.E., Lajolo F.M., Genovese M.I., Shetty K. (2010). Evaluation of Antiproliferative, Anti-Type 2 Diabetes, and Antihypertension Potentials of Ellagitannins from Strawberries (*Fragaria* × *ananassa* Duch.) Using In Vitro Models. J. Med. Food.

[27] Gao L., Wang X., Lin Z., Song N., Liu X., Chi X., Shao T. (2018). Antidiabetic and Neuroprotective Effect of the N-Butanol Extract of *Fragaria nilgerrensis* Schlecht. in STZ-Induced Diabetic Mice. Evid.-Based Complement. Altern. Med..

[28] Nugent T.C., Bibi A., Sadiq A., Shoaib M., Umar M.N., Tehrani F.N. (2012). Chiral picolylamines for Michael and aldol reactions: Probing substrate boundaries. Org. Biomol. Chem..

[29] Nugent T.C., Negru D.E., El-Shazly M., Hu D., Sadiq A., Bibi A., Umar M.N. (2011). Sequential Reductive Amination-Hydrogenolysis: A One-Pot Synthesis of Challenging Chiral Primary Amines. Adv. Synth. Catal..

[30] Sadiq A., Mahnashi M.H., Alyami B.A., Alqahtani Y.S., Alqarni A.O., Rashid U. (2021). Tailoring the substitution pattern of pyrrolidine-2,5-dione for discovery of new structural template for dual COX/LOX inhibition. Bioorg. Chem..

[31] Nugent T.C., Sadiq A., Bibi A., Heine T., Zeonjuk L.L., Vankova N., Bassil B.S. (2012). Noncovalent Bifunctional Organocatalysts: Powerful Tools for Contiguous Quaternary-Tertiary Stereogenic Carbon Formation, Scope, and Origin of Enantioselectivity. Chem. Eur. J..

[32] Jabeen M., Choudhry M.I., Miana G.A., Rahman K.M., Rashid U., Khan H.-U., Arshia, Sadiq A. (2018). Synthesis, pharmacological evaluation and docking studies of progesterone and testosterone derivatives as anticancer agents. Steroids.

[33] Jan M.S., Ahmad S., Hussain F., Ahmad A., Mahmood F., Rashid U., Abid O.-U., Ullah F., Ayaz M., Sadiq A. (2019). Design, synthesis, in-vitro, in-vivo and in-silico studies of pyrrolidine-2,5-dione derivatives as multitarget anti-inflammatory agents. Eur. J. Med. Chem..

[34] Ahmad S., Iftikhar F., Ullah F., Sadiq A., Rashid U. (2016). Rational design and synthesis of dihydropyrimidine based dual binding site acetylcholinesterase inhibitors. Bioorg. Chem..

[35] Ahmad G., Rasool N., Rizwan K., Imran I., Zahoor A.F., Zubair M., Sadiq A., Rashid U. (2019). Synthesis, in-vitro cholinesterase inhibition, in-vivo anticonvulsant activity and in-silico exploration of *N*-(4-methylpyridin-2-yl)thiophene-2-carboxamide analogs. Bioorg. Chem..

[36] Sadiq A., Zeb A., Ullah F., Ahmad S., Ayaz M., Rashid U., Muhammad N. (2018). Chemical Characterization, Analgesic, Antioxidant, and Anticholinesterase Potentials of Essential Oils from *Isodon rugosus* Wall. ex. Benth. Front. Pharmacol..

[37] Munir A., Khushal A., Saeed K., Sadiq A., Ullah R., Ali G., Ashraf Z., Mughal E.U., Jan M.S., Rashid U. (2020). Synthesis, in-vitro, in-vivo anti-inflammatory activities and molecular docking studies of acyl and salicylic acid hydrazide derivatives. Bioorg. Chem..

[38] Farooq U., Naz S., Shams A., Raza Y., Ahmed A., Rashid U., Sadiq A. (2019). Isolation of dihydrobenzofuran derivatives from ethnomedicinal species *Polygonum barbatum* as anticancer compounds. Biol. Res..

[39] Sultana N., Sarfraz M., Tanoli S.T., Akram M.S., Sadiq A., Rashid U., Tariq M.I. (2017). Synthesis, crystal structure determination, biological screening and docking studies of N^1^-substituted derivatives of 2,3-dihydroquinazolin-4(1*H*)-one as inhibitors of cholinesterases. Bioorg. Chem..

[40] American Diabetes Association (2022). Introduction: Standards of Medical Care in Diabetes—2022. Diabetes Care.

[41] Takács I., Szekeres A., Takács Á., Rakk D., Mézes M., Polyák Á., Lakatos L., Gyémánt G., Csupor D., Kovács K.J. (2020). Wild Strawberry, Blackberry, and Blueberry Leaf Extracts Alleviate Starch-Induced Hyperglycemia in Prediabetic and Diabetic Mice. Planta Med..

[42] Kumar S., Narwal S., Kumar V., Prakash O. (2011). α-glucosidase inhibitors from plants: A natural approach to treat diabetes. Pharmacogn. Rev..

[43] Scalliet G., Journot N., Jullien F., Baudino S., Magnard J.-L., Channelière S., Vergne P., Dumas C., Bendahmane M., Cock J. (2002). Biosynthesis of the major scent components 3,5-dimethoxytoluene and 1,3,5-trimethoxybenzene by novel rose *O*-methyltransferases. FEBS Lett..

[44] Ferreira L.A., Henriques O.B., Andreoni A.A., Vital G.R., Campos M.M., Habermehl G.G., de Moraes V.L. (1992). Antivenom and biological effects of ar-turmerone isolated from *Curcuma longa* (Zingiberaceae). Toxicon.

[45] Golding B.T., Pombo E., Samuel C.J. (1982). Turmerones: Isolation from turmeric and their structure determination. J. Chem. Soc. Chem. Commun..

[46] Jankasem M., Wuthi-Udomlert M., Gritsanapan W. (2013). Antidermatophytic Properties of *Ar*-Turmerone, Turmeric Oil, and *Curcuma longa* Preparations. ISRN Dermatol..

[47] Saga Y., Hatakenaka Y., Matsumoto M., Yoshioka Y., Matsumura S., Zaima N., Konishi Y. (2020). Neuroprotective effects of aromatic turmerone on activity deprivation-induced apoptosis in cerebellar granule neurons. NeuroReport.

[48] Wang S.Y., Lin H.-S. (2000). Antioxidant Activity in Fruits and Leaves of Blackberry, Raspberry, and Strawberry Varies with Cultivar and Developmental Stage. J. Agric. Food Chem..

[49] Scalzo J., Mezzetti B., Battino M. (2005). Total antioxidant capacity evaluation: Critical steps for assaying berry antioxidant features. BioFactors.

[50] Tulipani S., Romandini S., Busco F., Bompadre S., Mezzetti B., Battino M. (2009). Ascorbate, not urate, modulates the plasma antioxidant capacity after strawberry intake. Food Chem..

[51] Kiselova Y., Ivanova D., Chervenkov T., Gerova D., Galunska B., Yankova T. (2006). Correlation between the in vitro antioxidant activity and polyphenol content of aqueous extracts from bulgarian herbs. Phytother. Res..

[52] Cheplick S., Kwon Y.-I., Bhowmik P., Shetty K. (2010). Phenolic-linked variation in strawberry cultivars for potential dietary management of hyperglycemia and related complications of hypertension. Bioresour. Technol..

[53] El-Hawary S.S., Mohammed R., El-Din M.E., Hassan H.M., Ali Z.Y., Rateb M.E., El Naggar E.M.B., Othman E.M., Abdelmohsen U.R. (2021). Comparative phytochemical analysis of five Egyptian strawberry cultivars (*Fragaria × ananassa* Duch.) and antidiabetic potential of Festival and Red Merlin cultivars. RSC Adv..

[54] Mandave P., Khadke S., Karandikar M., Pandit V., Ranjekar P., Kuvalekar A., Mantri N. (2017). Antidiabetic, Lipid Normalizing, and Nephroprotective Actions of the Strawberry: A Potent Supplementary Fruit. Int. J. Mol. Sci..

[55] Ibrahim D.S., El-Maksoud M.A.E.A. (2015). Effect of strawberry (*Fragaria* × *ananassa*) leaf extract on diabetic nephropathy in rats. Int. J. Exp. Pathol..

[56] Moazen S., Amani R., Rad A.H., Shahbazian H., Ahmadi K., Jalali M.-T. (2013). Effects of Freeze-Dried Strawberry Supplementation on Metabolic Biomarkers of Atherosclerosis in Subjects with Type 2 Diabetes: A Randomized Double-Blind Controlled Trial. Ann. Nutr. Metab..

[57] Shah S.M.M., Ahmad Z., Yaseen M., Shah R., Khan S., Khan B. (2015). Phytochemicals, in vitro antioxidant, total phenolic contents and phytotoxic activity of *Cornus macrophylla* Wall bark collected from the North-West of Pakistan. Pak. J. Pharm. Sci..

[58] Shah S.M.M., Ullah F., Shah S.M.H., Zahoor M., Sadiq A. (2012). Analysis of chemical constituents and antinociceptive potential of essential oil of *Teucrium Stocksianum* bioss collected from the North West of Pakistan. BMC Complement. Altern. Med..

[59] Aslam H., Khan A.-U., Naureen H., Ali F., Ullah F., Sadiq A. (2018). Potential application of *Conyza canadensis* (L) Cronquist in the management of diabetes: In vitro and in vivo evaluation. Trop. J. Pharm. Res..

[60] Sadiq A., Mahnashi M.H., Rashid U., Jan M.S., Alshahrani M.A., Huneif M.A. (2022). 3-(((1*S*,3*S*)-3-((*R*)-Hydroxy(4-(trifluoromethyl)phenyl)methyl)-4-oxocyclohexyl)methyl)pentane-2,4-dione: Design and Synthesis of New Stereopure Multi-Target Antidiabetic Agent. Molecules.

[61] Huneif M.A., Alshehri D.B., Alshaibari K.S., Dammaj M.Z., Mahnashi M.H., Majid S.U., Javed M.A., Ahmad S., Rashid U., Sadiq A. (2022). Design, synthesis and bioevaluation of new vanillin hybrid as multitarget inhibitor of α-glucosidase, α-amylase, PTP-1B and DPP4 for the treatment of type-II diabetes. Biomed. Pharmacother..

[62] Mahnashi M.H., Alyami B.A., Alqahtani Y.S., Jan M.S., Rashid U., Sadiq A., Alqarni A.O. (2021). Phytochemical profiling of bioactive compounds, anti-inflammatory and analgesic potentials of *Habenaria digitata* Lindl.: Molecular docking based synergistic effect of the identified compounds. J. Ethnopharmacol..

