# Supplementary material for: α-Glucosidase, α-Amylase and Antioxidant Evaluations of Isolated Bioactives from Wild Strawberry
Source: Molecules. 2022 May 26;27(11):3444. doi: 10.3390/molecules27113444 (PMC9182347; doi:10.3390/molecules27113444)
Supplement: Supplementary file 1 [file molecules-27-03444-s001.zip › molecules-1704697-supplementary.pdf]

molecules

MDPI

Supplementary Materials

α-Glucosidase, α-Amylase and Antioxidant Evaluations of Isolated Bioactives from Wild Strawberry

Mohammed A. Huneif [1], Seham M. Alqahtani [1], Alqahtani Abdulwahab [1], Sultan A. Almedhesh [1], Mater H. Mahnashi [2,*], Muhammad Riaz [3], Najm Ur-Rahman [3], Muhammad Saeed Jan [4], Farhat Ullah [5], Muhammad Aasim [6] and Abdul Sadiq [5,*]

1 Pediatric Department, Medical College, Najran University, Najran 55461, Saudi Arabia; huneif@hotmail.com (M.A.H.); drseham2015@gmail.com (S.M.A.); aaalsharih@nu.edu.sa (A.A.); almedhesh31@hotmail.com (S.A.A.)
2 Department of Pharmaceutical Chemistry, College of Pharmacy, Najran University, Najran 55461, Saudi Arabia
3 Department of Pharmacy, Shaheed Benazir Bhutto University, Sheringal 18050, Pakistan; pharmariaz@gmail.com (M.R.); najm@sbbu.edu.pk (N.U.-R.)
4 Department of Pharmacy, University of Swabi, Swabi, Pakistan; saeedjanpharmacist@gmail.com
5 Department of Pharmacy, Faculty of Biological Sciences, University of Malakand, Chakdara 18000, Pakistan; farhataziz80@hotmail.com
6 Department of Biotechnology, Faculty of Biological Sciences, University of Malakand, Chakdara 18000, Pakistan; draasim@uom.edu.pk
* Correspondence: mhmahneshi@nu.edu.sa (M.H.M.); sadiquom@yahoo.com (A.S.); Tel.: +96-65-0873-4539 (M.H.M.); +92-03-01-229-7102 (A.S.)

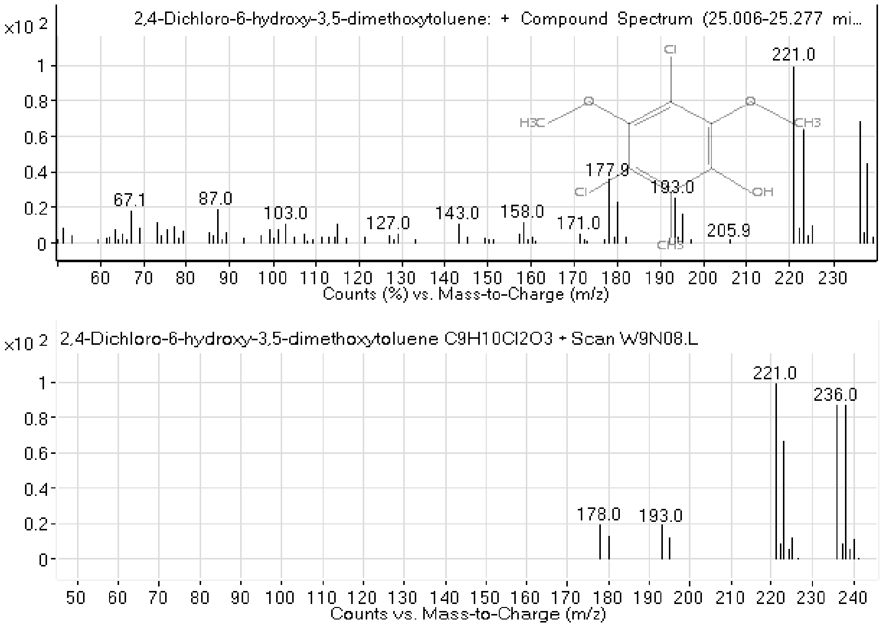

Figure S1. GC-MS chromatogram of Compound **1**.

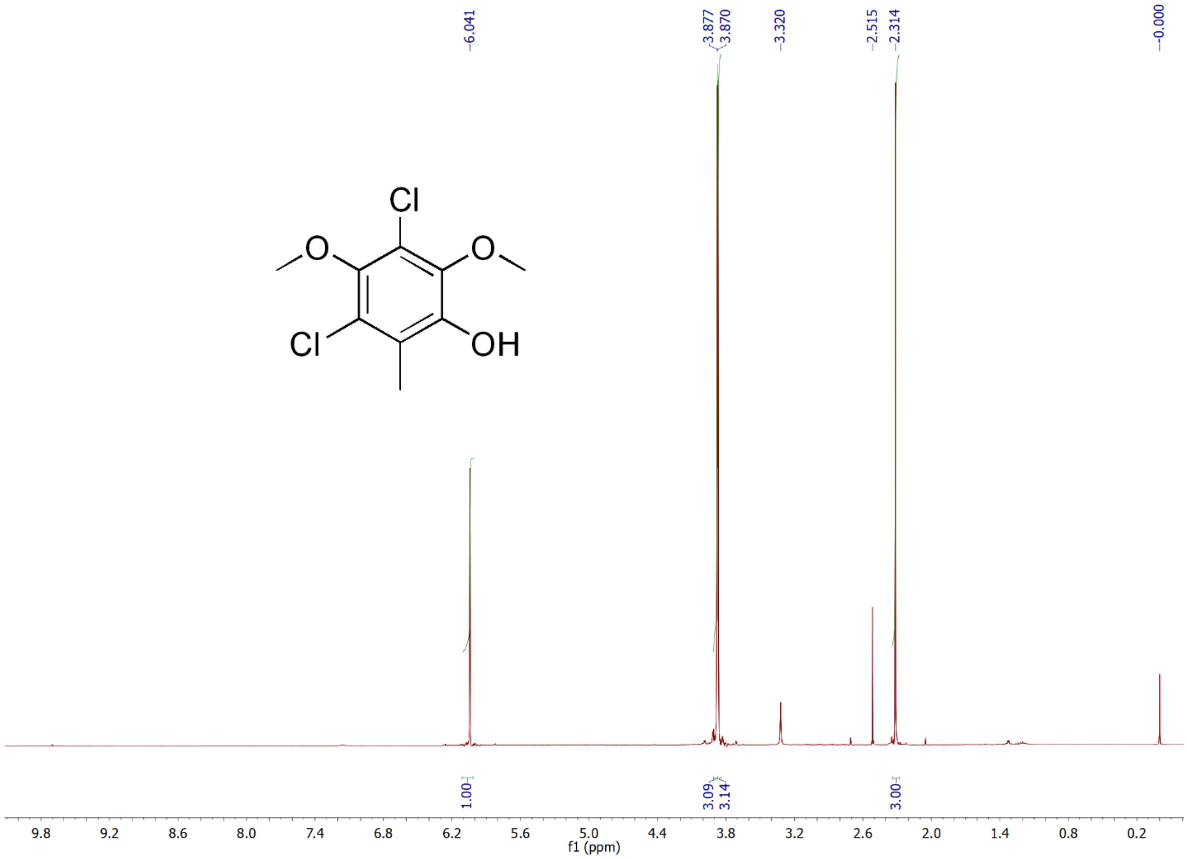

Figure S2. ^{1}H-NMR spectrum of Compound **1**.

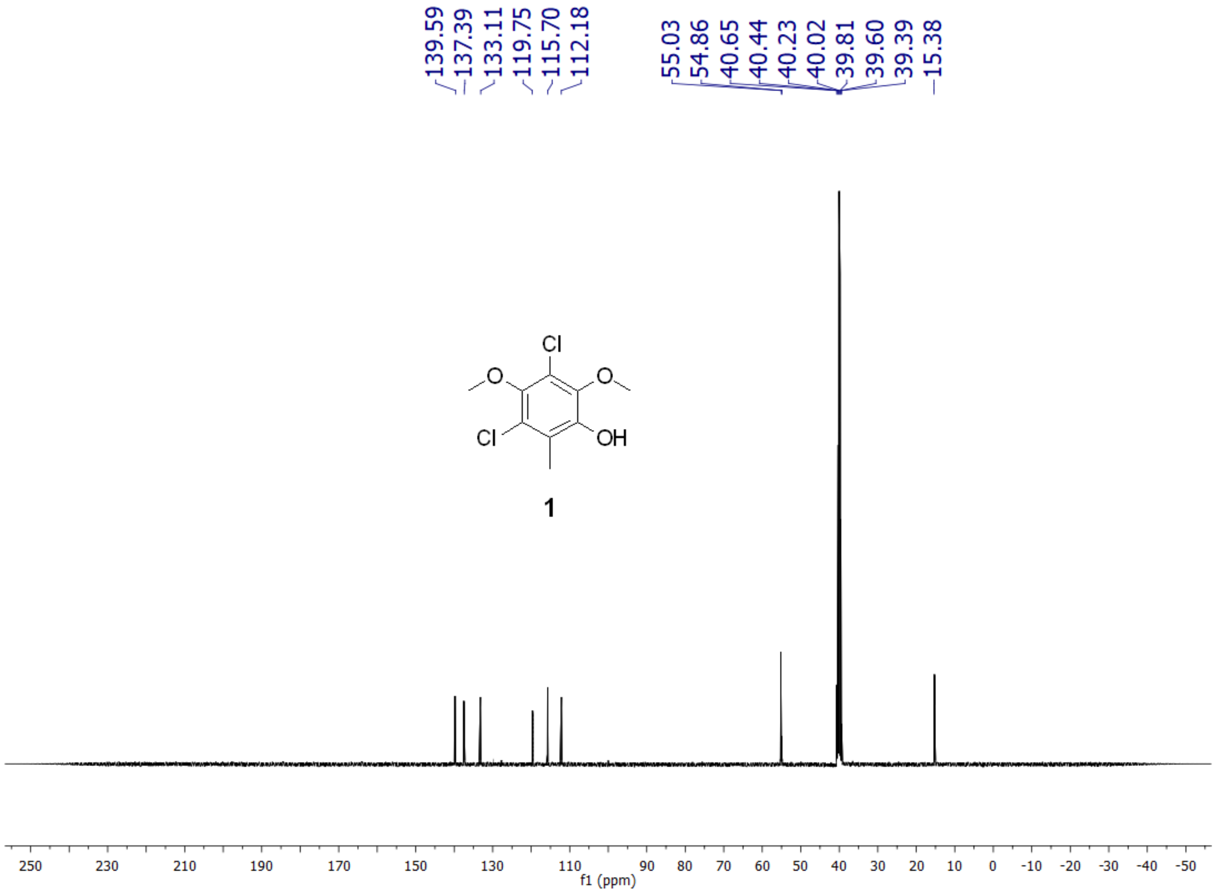

Figure S3. ^{13}C-NMR spectrum of Compound **1**.

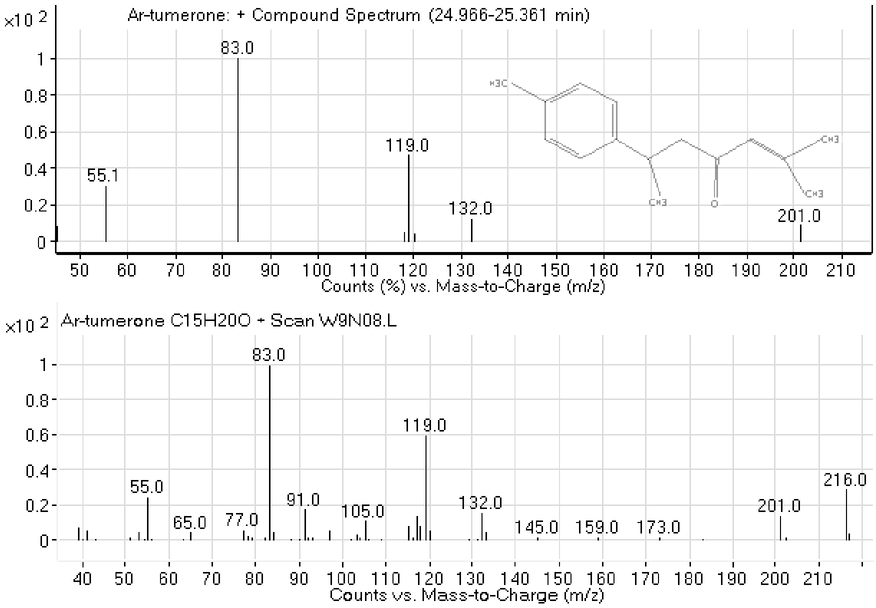

Figure S4. GC-MS chromatogram of Compound **2**.

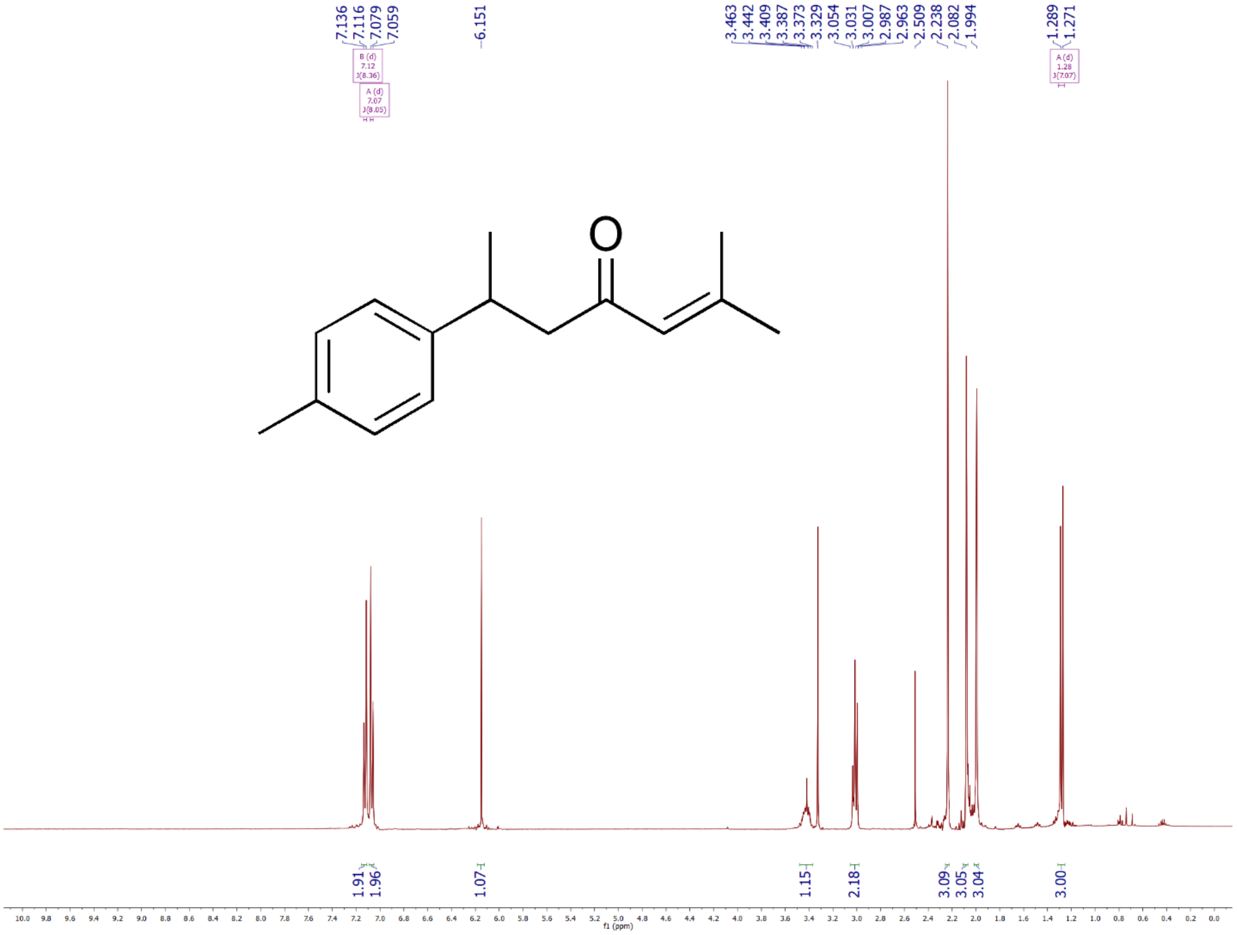

Figure S5. ^{1}H-NMR spectrum of Compound **2**.

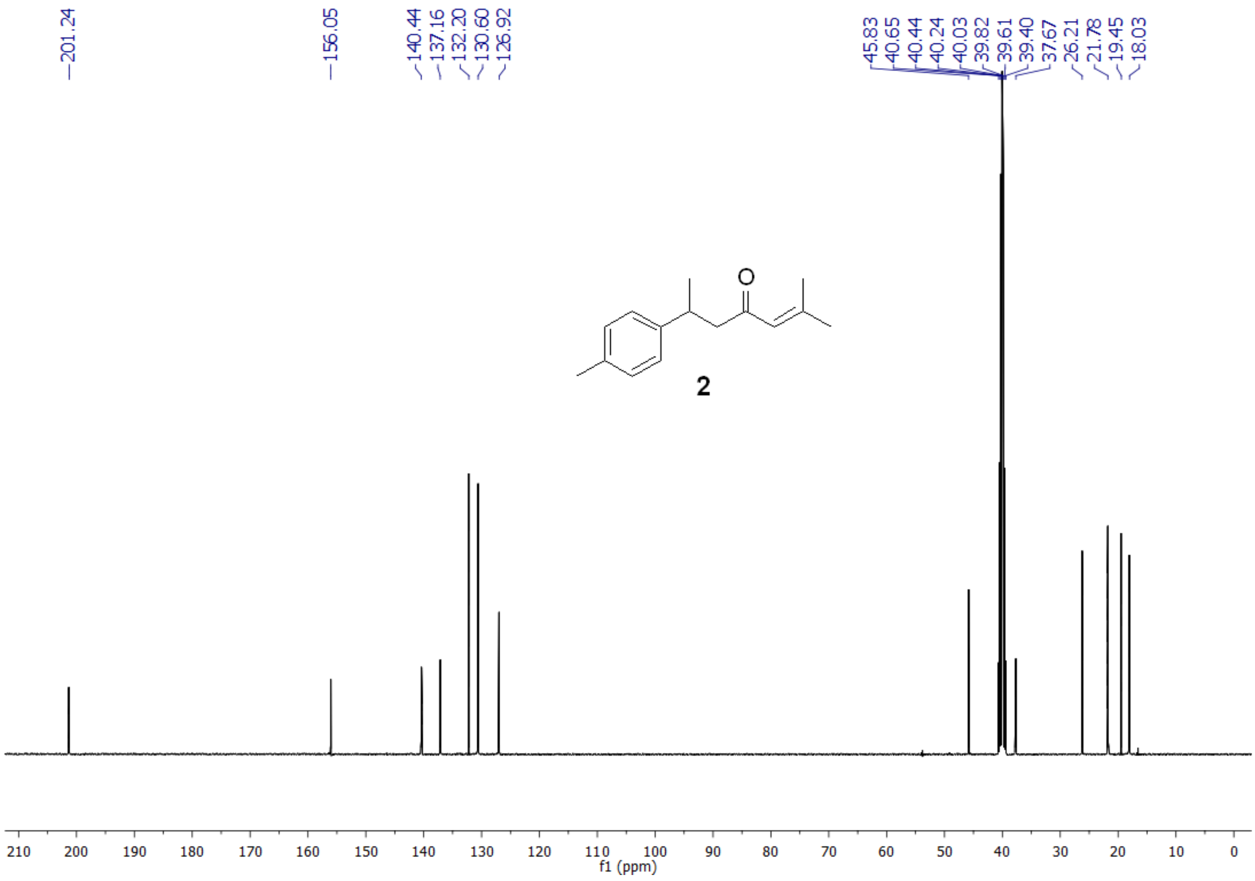

Figure S6. ^{13}C-NMR spectrum of Compound **2**.
